# Expression and Function Studies of *CYC*/*TB1*-Like Genes in the Asymmetric Flower *Canna* (Cannaceae, Zingiberales)

**DOI:** 10.3389/fpls.2020.580576

**Published:** 2020-12-04

**Authors:** Qianxia Yu, Xueyi Tian, Canjia Lin, Chelsea D. Specht, Jingping Liao

**Affiliations:** ^1^Key Laboratory of Plant Resources Conservation and Sustainable Utilization, South China Botanical Garden, Chinese Academy of Sciences, Guangzhou, China; ^2^College of Life Sciences, University of Chinese Academy of Science, Beijing, China; ^3^Guangdong Provincial Key Laboratory of Digital Botanical Garden, South China Botanical Garden, Chinese Academy of Sciences, Guangzhou, China; ^4^School of Integrative Plant Science, Section of Plant Biology and the L.H. Bailey Hortorium, Cornell University, Ithaca, NY, United States; ^5^Center of Conservation Biology/Economic Botany/Plant Ecology, Core Botanical Gardens, Chinese Academy of Sciences, Guangzhou, China

**Keywords:** Canna indica, floral asymmetry, Zingiberales, CYCLOIDEA, TEOSINTE BRANCHED1

## Abstract

The asymmetric flower, lacking any plane of symmetry, is rare among angiosperms. *Canna indica* L. has conspicuously asymmetric flowers resulting from the presence of a half-fertile stamen, while the other androecial members develop as petaloid staminodes or abort early during development. The molecular basis of the asymmetric distribution of fertility and petaloidy in the androecial whorls remains unknown. Ontogenetic studies have shown that *Canna* flowers are borne on monochasial (cincinnus) partial florescences within a racemose inflorescence, with floral asymmetry likely corresponding to the inflorescence architecture. Given the hypothesized role of *CYC*/*TB1* genes in establishing floral symmetry in response to the influence of the underlying inflorescence architecture, the spatiotemporal expression patterns of three *Canna CYC*/*TB1* homologs (*CiTBL1a*, *CiTBL1b-1*, and *CiTBL1b-2*) were analyzed during inflorescence and floral development using RNA *in situ* hybridization and qRT-PCR. In the young inflorescence, both *CiTBL1a* and *CiTBL1b-1* were found to be expressed in the bracts and at the base of the lateral florescence branches, whereas transcripts of *CiTBL1b-2* were mainly detected in flower primordia and inflorescence primordia. During early flower development, expression of *CiTBL1a* and *CiTBL1b-1* were both restricted to the developing sepals and petals. In later flower development, expression of *CiTBL1a* was reduced to a very low level while *CiTBL1b-1* was detected with extremely high expression levels in the petaloid androecial structures including the petaloid staminodes, the labellum, and the petaloid appendage of the fertile stamen. In contrast, expression of *CiTBL1b-2* was strongest in the fertile stamen throughout flower development, from early initiation of the stamen primordium to maturity of the ½ anther. Heterologous overexpression of *CiTBL* genes in *Arabidopsis* led to dwarf plants with smaller petals and fewer stamens, and altered the symmetry of mature flowers. These data provide evidence for the involvement of *CYC*/*TB1* homologs in the development of the asymmetric Cannaceae flower.

## Introduction

Floral symmetry, one of the major features defining the shape of a flower, has been a special focus of botanists for many years. According to the number of symmetry planes, three main types of floral symmetry are recognized: actinomorphy (with several symmetry planes; i.e., polysymmetry or radial symmetry), zygomorphy (with one symmetry plane; i.e., monosymmetry or bilateral symmetry), and asymmetry (no symmetry plane; Endress, 1999; Citerne et al., 2010; Hileman, 2014). Actinomorphy is considered as the ancestral state of flowering plants and it appears in many clades, especially the early angiosperm lineages (Sauquet et al., 2017). Zygomorphic flowers evolved relatively late in the fossil record (Turonian, Upper Cretaceous, ca. 90 mya), and zygomorphy is considered a derived trait relative to actinomorphy (Gandolfo et al., 1998). Evolution of floral zygomorphy is thought to facilitate plant–pollinator interactions by enhancing pollination specificity and success (Neal et al., 1998). Zygomorphy is dominant in some species-rich lineages such as Orchidaceae, Fabaceae, and Lamiales (Endress, 2012). Floral asymmetry, like floral zygomorphy, appears in different forms that are not all homologous and can involve different floral organs and organ whorls. Compared with actinomorphic or zygomorphic flowers, asymmetric flowers are much less common across the angiosperms, and the genetic and molecular mechanisms underlying asymmetry have not been studied in detail as they have for zygomorphy (Endress, 2012).

**Figure 1:**
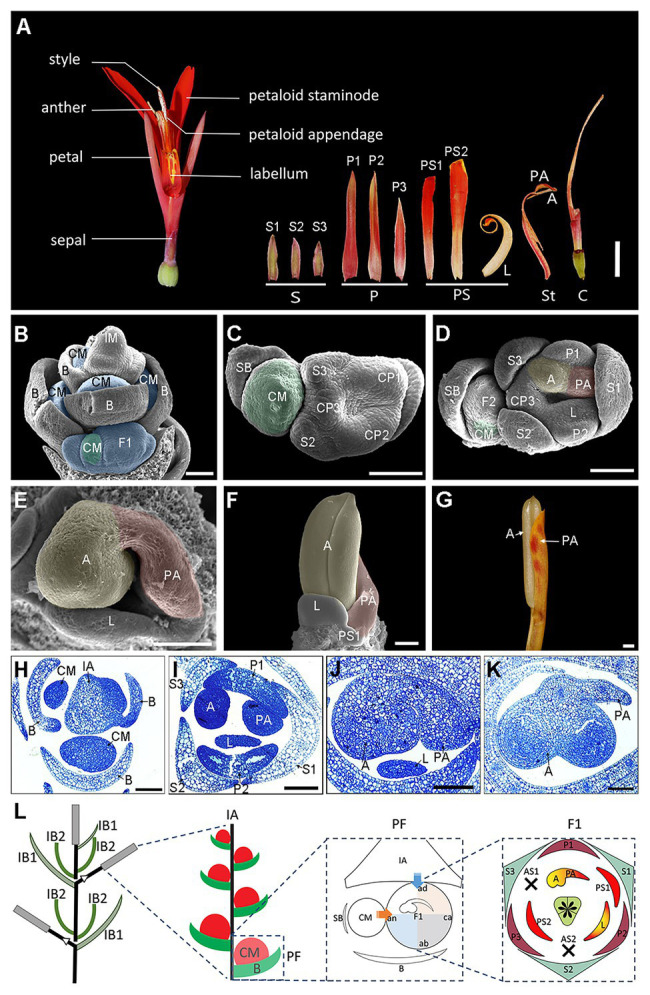
Flower anatomy and ontogeny of *Canna indica*. **(A)** Flower dissection showing the floral structure. **(B–F)** Flower organogenesis observed under a scanning electronic microscopy. **(B)** Lateral view of a young inflorescence with developing cincinni meristems subtended by the primary bracts. **(C)** Top view of a cincinni. Three sepal primordia and three petal-stamen common primordia have already formed in the primary flower. **(D)** Further development of the cincinni. CP1 separates into the first petal and the adaxial stamen, and the later one develops into an anther primordium in the left side and a petaloid appendage primordium in the right side. CP2 separates into the second petal and the labellum primordium. The second floral meristem has formed, and the cincinnus meristem moves to the abaxial side of the secondary flower. **(E)** Close-up of the asymmetric pattern of the adaxial stamen in the primary flower. The anther primordium is growing quickly while the petaloid appendage slows down its growing. **(F)** Front view of a fully developed anther. The petaloid appendage continues to develop in a petaloid way together with other petaloid staminodes (PS2 is not shown in this figure). **(G)** A fertile stamen before mature. **(H–K)** Transverse semithin sections stained with toluidine blue representing the same developmental stages with **(B,D,E,F)**, respectively. **(L)** Illustrated inflorescence and floral diagram. The arrows and shades in blue and orange represent the presumed impact on the primary flower by the main florescence axis and the cincinnus meristem, respectively. A, anther; ab, abaxial; ad, adaxial; an, anodic; AS, aborted staminode; B, primary bract; C, carpel; ca, cathodic; CM, cincinni meristem; CP, petal-stamen common primordium; FM, flower meristem; IA, florescence axis; IB1, the bract enclosing the primary rachis of the inflorescence; IB2, the bract which are placed at the base of the new rachis at each branching point, enclosing the new rachis. IM, inflorescence meristem; L, labellum; P, petal; PA, petaloid appendage; PF, partial florescence; PS, petaloid staminode; S, sepal; SB, secondary bract. Blue shades in **(B)** indicate partial florescences enclosed by the primary bracts. Green shades in **(B–D)** indicate cincinnus meristems. Yellow and red shades in **(D–F)** indicate the anther primordium and the petaloid appendage primordium, respectively. Bars: **(A)** = 1 cm; **(B–K)** = 100 *μ*m.

Cannaceae (Zingiberales) is one of the few families that possesses truly asymmetric flowers. Ten species were recognized in Cannaceae, all of them placed in the single genus *Canna* (Maas-van de Kamer and Maas, 2008; Prince, 2010). The asymmetry of the *Canna* flower is mostly associated with an asymmetric distribution and elaboration of petaloidy and organ abortion across the six members of the androecium. *Canna* flowers consist of two androecial whorls, each with three members (Figure 1A). In the outer whorl, one androecial member develops into a mature petaloid staminode (i.e., infertile stamen) whereas the other two primordia typically abort shortly after initiation (Kirchoff, 1983; Miao et al., 2014). The inner whorl contains two petaloid staminodes plus the fertile stamen, which is in fact a “half-fertile stamen” comprising an anther with a single theca and a petaloid appendage (Kirchoff, 1983; Almeida et al., 2013; Fu et al., 2014; Miao et al., 2014; Tian et al., 2016, 2018). Of the two inner whorl staminodes, one is curved and referred to as the “labellum”. Both the half-fertile stamen and the presence of the labellum contribute to the asymmetry of the androecium. The style is also laminar (Morioka et al., 2015), making the gynoecium appear asymmetric as well. The presence of asymmetry across several floral whorls makes *Canna* a good taxon to study the genetic mechanisms involved in the development and elaboration of floral asymmetry.

*CYCLOIDEA*/*TEOSINTE BRANCHED1* (*CYC*/*TB1*)-like genes, a group of class II TCP transcription factors, are implicated in the evolution of key morphological traits (Cubas et al., 1999a; Howarth and Donoghue, 2006; Martin-Trillo and Cubas, 2010). *CYC* was first described in the zygomorphic flower of *Antirrhinum majus* and is now widely accepted as the main floral symmetry gene. Research in *Antirrhinum* revealed that *CYC* can respond to a common dorsoventral prepattern of the flowers in the lateral position of a raceme (Clark and Coen, 2002). The *CYC* gene is expressed in the dorsal region of the zygomorphic floral meristem and functions in stamen abortion and the regulation of petal growth by affecting the rate of cell division (Luo et al., 1996; Gaudin et al., 2000). Numerous studies across the angiosperms revealed that *CYC* homologs are expressed in the dorsal part of the lateral floral meristem in many of the major clades containing zygomorphic flowers (Martin-Trillo and Cubas, 2010; Hileman, 2014; Spencer and Kim, 2018). However, expression of a *CYC*/*TB1*-like gene (at least as one copy) was found on the ventral side of *Commelina* (Commelinales; Preston and Hileman, 2012), *Heliconia*, and *Costus* (Zingiberales; Bartlett and Specht, 2011) flowers, suggesting there may be a divergent mechanism specifying floral symmetry in the commelinid monocots.

*Canna* flowers are typically situated in an inflorescence that is considered a sympodial thyrse, with monochasial (cymose) terminal units or “partial florescences” subtended by the primary bracts of the racemose main florescence. Our previous observations found that when the partial florescence transformed into a single flower or a dichasial cyme, the flower of that modified florescence develops as zygomorphic due to changes in the degree of stamen petaloidy (Yu et al., in review). Those aberrant zygomorphic flowers suggest that the asymmetry of the normal *Canna* flower is strongly correlated with inflorescence structure (Yu et al., in review). It is proposed that *CYC*/*TB1*-like genes may respond to the asymmetric inflorescence system, resulting in their asymmetric expression in the floral primordium and leading to the asymmetric form of the mature flower.

In this research, we chose *C. indica* as a system to study the developmental mechanisms of floral asymmetry from both morphogenic and genetic aspects. Morphological and anatomical patterns were observed throughout the establishment of floral asymmetry *via* SEM and histological sectioning following the ontogeny of individual flowers and their inflorescences. *CYC*/*TB1* homologous genes in *C. indica* were identified and RNA *in situ* hybridization and qRT-PCR were carried out to investigate the spatiotemporal expression patterns of gene expression during inflorescence and floral development. Finally, we observed the *Arabidopsis* overexpression lines for three *Canna*-specific *CYC*/*TB1* genes (*CiTBL1a*, *CiTBL1b-1*, and *CiTBL1b-2*) to characterize the possible functions of these genes during plant development. Altogether, our work in *C. indica* is the first attempt to propose a mechanism for the origins of floral asymmetry in this lineage, and results will provide a foundation for studying the genetic basis controlling floral asymmetry across flowering plants.

## Materials and Methods

### Plant Materials and Growth Conditions

All the plant materials of *C. indica* used in this study were collected from the Ginger Garden and Aquatic Plants Section of South China Botanical Garden, Guangzhou, China.

*Arabidopsis thaliana* seeds were sterilized and germinated on MS medium (pH 5.8). The seedlings were transplanted to pots containing the mixture of vermiculite and Pindstrup substrate (Pindstrup, Ryomgaad, Denmark; 1:1), and grown to flowering stage in a growth chamber at 22°C with 16-h light/8-h dark cycles. All *Arabidopsis* transgenic lines were in the Columbia background, which was also used as the wild-type control.

### Scanning Electron Microscopy (SEM)

Inflorescence and floral tissues at different developmental stages fixed with FAA were dissected as necessary to reveal internal floral organs, and then dehydrated in an alcohol series. Specimens were critical point dried with CO_2_, mounted on stubs, sputter-coated with gold, and observed under a JSM-6360LV scanning electron microscope (JEOL, Tokyo, Japan). Micrographs were adjusted for brightness, contrast, and color balance using Adobe Photoshop CC (Adobe, San Jose, CA, USA).

### Histology

Inflorescence and floral tissues were dissected and stored in 2% glutaraldehyde in 0.1 M phosphate buffer at pH 7.2–7.4 overnight at 4°C. The specimens were washed six times in phosphate-buffered saline (PBS) and postfixed in 1% w/v osmium tetroxide for 4 h, and then washed six times in PBS for 2 h. Samples were dehydrated in a graded ethanol series, embedded in Spurr resin, and sectioned at 2 *μ*m with glass knives on a LKB-11800 microtome. Sections were stained with toluidine blue and observed under a Leica DFC550 Microscopy (Wetzlar, Germany).

### Gene Retrieval, Protein Sequence Alignment, and Phylogenetic Analysis

The sequences of *CYC*/*TB1* homologous gene were retrieved from the *C. indica* inflorescence transcriptome we generated before (Tian et al., 2016) by BLAST similarity searches. Sequence information for the four *CiTBL* genes is available in GenBank under accession numbers MW176099 to MW176102.

To affirm they are in the *CYC*/*TB1* subclade of TCP family, phylogenetic analysis was carried out using MEGA version 6.06 by the full length of protein sequences from GenBank according to Luo et al. (1996, 1999), Doebley et al. (1997), Takeda et al. (2003), Yuan et al. (2009), Martin-Trillo and Cubas (2010), Lin et al. (2016), and Chai et al. (2017). Distance matrices used the PAM-Dayhoff model of amino acid substitution; the phylogenetic tree was constructed with the maximum-likelihood method, and bootstrap analyses used 1,000 resampling replicates. The Zingiberales TBL ML tree was re-conducted according to Bartlett and Specht (2011). Additional sequences of *C. glauca* were retrieved from the unpublished transcriptome, and sequences of *Musa acuminate* subsp. *malaccensis* were retrieved from The Banana Genome Hub (D’Hont et al., 2012).^1^

Putative protein sequence alignment of *CiTBL*s and *Antirrhinum CYC* was conducted by Clustal X2 with default settings.

### Quantitative Real-Time RT-PCR

Total RNA was extracted from young flowers of three different stages (1.0, 1.5, and 2.0 cm in length) and seven different organs (petal, petaloid staminode, labellum, anther, petaloid appendage, carpel, and leaf) using RNAprep Pure Kit (Tiangen, Beijing, China). All the floral organs dissected are half the length of the mature organ to make sure they are in rapid growth. About 3 *μ*g total RNA was reversely transcribed to the first strand of the cDNA in a 20-μl volume using EasyScript One-Step gDNA Removal and cDNA Synthesis SuperMix (TransGen, Beijing, China) with oligo (dT) primers. qRT-PCR was performed using specific primer pairs (Supplementary Table S1) under the manual of LightCycler 480 Real-Time PCR System (Roche, Basel, Switzerland). Each qPCR reaction (total volume of 20 μl) contains 10 μl of Top Green qPCR SuperMix (TransGen, Beijing, China), 0.2 μl of each primer, 1 μl of 1:5 diluted cDNA, and 8.2 μl ddH_2_O. Thermal cycling consisted of a hold at 94°C for 30 s, followed by 40 cycles of 94°C for 5 s and 60°C for 30 s. The temperature was then gradually raised by 0.5°C every 10 s for performing melting curve analysis. Three biological replicates were performed with three technical replicates for each sample. The *Δ*ΔCt method was employed with *CiActin* and *CiPP2A* (*protein phosphatase 2A*) as endogenous controls (Livak and Schmittgen, 2001).

### RNA *in situ* Hybridization

To generate specific digoxigenin-labeled riboprobes, nonconservative region of *CiTBL1a* (291 nt), *CiTBL1b-1* (256 nt), and *CiTBL1b-2* (244 nt) were amplified with primers listed in Supplementary Table S1 and cloned into pGEM-T vector (Promega Fitchburg, WI, USA) following manufacturer instructions. *In vitro* transcription was performed using SP6/T7 DIG RNA Labeling Kit (Roche, Basel, Switzerland). Inflorescence and floral tissues were fixed, sectioned, and hybridized to the probes as described previously (Tian et al., 2016, 2018). The hybridized sections were visualized under brightfield illumination with a Leica DFC550 Microscope (Wetzlar, Germany).

### Construction of *CiTBL* Transgenic Plants in *Arabidopsis*

The ORF of *CiTBL1a*, *CiTBL1b-1*, and *CiTBL1b-2* were amplified using the primer pairs listed in Supplementary Table S1. The overexpression construct of *CiTBL* allele, *35S::CiTBL1a*, *35S::CiTBL1b-1*, and *35S::CiTBL1b-2* was engineered to pCAMBIA1302 using One Step Cloning Kit (Vazyme, Nanjing, China). The sequence confirmed vectors were then transformed into *Agrobacterium* strain GV3101. The *Arabidopsis* floral dip transformation protocol was followed by Clough and Bent (1988). The floral dip procedure was repeated once again after a week for obtaining more transformed seeds. Two to five putative transgenic *A. thaliana* T1 seedlings were selected for each construction by antibiotic hygromycin and PCR confirmed (2 T1 lines for *35S, CiTBL1a*, 5 T1 lines for *35S::CiTBL1b-1*, and 3 T1 lines for *35S::CiTBL1b-2*). Several representative T1 plants with obvious phenotypes were selfed into T2 plants to document the phenotypic effect of each construct. The mRNA expression level of *CiTBL1a*, *CiTBL1b-1*, and *CiTBL1b-2* in flowers of at least five independent T2 transgenic plants was verified by semi-quantitative RT-PCR. *AtActin* was chosen as a reference gene and amplified with 30 cycles using primers AtActinF/R (Supplementary Table S1). For each allele construction, 15–20 independent T2 transgenic individuals of overexpressing *CiTBL1a*, *CiTBL1b-1*, and *CiTBL1b-2*, together with T1 plants, were summarized for the phenotypic effects on plant form and floral morphology. The images of petal epidermal cells were taken under a Leica DVM6 Digital Microscope (Wetzlar, Germany). To measure the cell size of the adaxial epidermal petal cells, the middle regions of the petals were zoomed in and the cell numbers in a 100 × 100 *μ*m^2^ square were counted.

## Results

### The Establishment of Floral Asymmetry in *Canna*

The inflorescence of *C. indica* is a terminal, sympodially branched thyrse, comprising repeated units of florescences. Each florescence is monochasial cyme/cincinnus (Figures 1B,H,L). Bracts (B) on the main florescence axis (IA) subtend a lateral cincinnus meristem (CM), which will ultimately give rise to one or two flowers (Figures 1B,H). In *Canna*, the secondary bract that subtends a terminal flower is present on the left (anodic, an) side of the primary flower in a polar view. This is the side of the flower that lies in the direction of the rise of the phyllotactic helix which is typically sinistrose, or left-handed, in *Canna*. The cathodic side (ca) is the side that lies opposite the rise of the phyllotactic helix. As the secondary flower (F2) often ceases development when it is still quite small, only the development of the primary flower (F1) is described for this study. The terms adaxial, abaxial, anodic, and cathodic all refer to the primary flower of a florescence unit that is considered a cincinnus.

In *Canna*, floral organs of each whorl develop in a clockwise sequence (Kirchoff, 1983; Almeida et al., 2013; Miao et al., 2014). Three sepal primordia initiate sequentially at the three angles of the floral apex. The individual sepal (S1) opposite to the cincinnus meristem arises first and it is larger than the other two at early stages (Figure 1C), with the sepals all becoming almost equal in size at maturity (Figure 1A). Three common stamen-petal primordia (part of the ring primordium) arise after sepal initiation (Figure 1C). The shape of floral apex is approximately zygomorphic, and the symmetry plane passes through the secondary bract and the cincinnus meristem, suggesting the monochasial partial inflorescence structure may affect the shape of the flower at a very early stage. As the common primordia widen, separation occurs between the petals (toward the outside) and inner androecial members (to the inside). The three petals are basally connate and unequal at maturity, with the lateral one remaining smaller than the other two (Figure 1A). The adaxial inner stamen primordium develops to two features – an anther (A) and a petaloid appendage (PA). To better elucidate the asymmetric development of the fertile stamen, we divided the continuous process into three main stages using several landmark events. In stage 1, the anther primordium and the petaloid appendage primordium are equal in size at initiation (Figures 1D,I). In stage 2, the petaloid appendage primordium slows in growth while the anther primordium develops relatively rapidly as a radial structure (Figures 1E,J). The anther is fully formed in stage 3, while the petaloid appendage primordium, which is accompanied by the labellum and other petaloid staminode primordia, continues to develop with a dorsiventrality to become a petaloid structure (Figures 1F,G,K).

In a *Canna* flower, the half-fertile stamen forms on the adaxial-anodic portion of the floral apex and is adjacent to both the florescence axis and the next older flower in a cincinnus (Figure 1L). The petaloid structures including the inner petaloid staminode, the labellum, and the petaloid appendage are either distal to the florescence axis or the cincinnus meristem, indicating that the fate of the meristem tissue in the androecial whorls is somewhat linked with the flower’s position within the inflorescence (Figure 1L).

### *CYC*/*TB1* Homologs in *C. indica*

**Figure 2:**
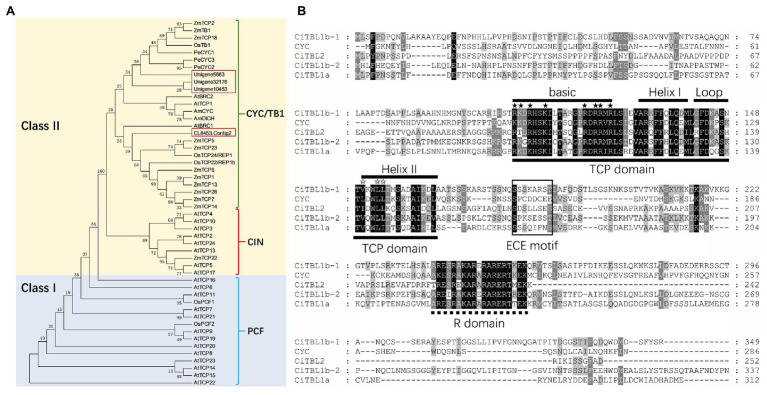
Phylogenetic analysis and protein sequences analysis of *CiTBLs*. **(A)** Unrooted phylogram of protein ML analysis with 1,000 bootstraps. All of the AtTCPs and AtBRCs are from *Arabidopsis*; AmCYC and AmDICH, *Antirrhinum*; OsTCPs, OsPCFs, and OsTB1, rice; ZmTCPs and ZmTB1, maize; PeCYCs, *Phalaenopsis equestris*. **(B)** Multiple alignment of putative protein sequences of CiTBL1a, CiTBL1b-1, CiTBL1b-2, and CYC. TCP domain is underlined with the solid line and R domain is underlined with the dotted line. ★ Indicates the conserved basic residues, and ☆ indicates the LXXLL motif.

Sequences of four putative *CYC*/*TB1* homologs, *CiTBL1a* (*Unigene32176_All*), *CiTBL1b-1* (*Unigene10453_All*), *CiTBL1b-2* (*Unigene5663_All*), and *CiTBL2* (*CL8453.Contig2*), were obtained from an existing transcriptome database (Tian et al., 2016). Phylogenetic analysis of protein sequences placed all four *CiTBL* genes into the CYC/TB1 clade of Class II TCP transcription factors. In this clade, the four *CiTBL* genes were clustered with *Antirrhinum CYC* and *DICH*, maize *TB1*, and *Arabidopsis TCP1*, *BRC1*, *BRC2*, demonstrating their homology with *CYC*/*TB1*-like genes (Figure 2A).

To better understand the evolutionary relationships of Zingiberales *CYC/TB1*-like genes, we combined the recovered *Canna* sequences with nucleotide sequences derived from a previous publication (Bartlett and Specht, 2011) and an unpublished transcriptome database for *C. indica* and *C. glauca*. The results of the phylogenetic analyses of this combined dataset generally conform to previous phylogenetic hypotheses for Zingiberales TB1 homologs, recovering three major clades (ZinTBL1a, ZinTBL1b, and ZinTBL2). In this study, the ZinTBL1b clade was further divided into two subclades: ZinTBL1b-1 and ZinTBL1b-2 (Supplementary Figure S1). Four TBL copies were found in both *C. indica* and *C. glauca* belonging to ZinTBL1a, ZinTBL1b-1, ZinTBL1b-2, and ZinTBL2 clades, respectively, suggesting that the gene duplication event leading to these four clades occurred before the divergence of modern species of Cannaceae.

The full-length ORFs of *CiTBL1a*, *CITBL1b-1*, *CiTBL1b-2*, and *CiTBL2* are 933, 1,047, 1,131, and 756 bp and encode putative proteins of 311, 349, 337, and 252 amino acids, respectively. Each protein contains a conserved 59-amino acid TCP domain, a 19-amino acid R domain, and a 7-amino acid ECE motif (Figure 2B). Sequence alignment shows the putative proteins of these four *CiTBL* genes share 34.05, 35.53, 35.96, and 30.87% similarity with *Antirrhinum* CYC protein, respectively. As to the TCP domain and R domain, CiTBL1a, CiTBL1b-1, CiTBL1b-2, and CiTBL2 share 84.62, 84.62, 83.88, and 79.49% amino acid sequence similarity with *Antirrhinum* CYC. The ECE motifs and the non-conserved intervening regions of CiTBLs and AmCYC are clearly divergent.

### Spatiotemporal Expression Patterns of *CiTBL1a*, *CiTBL1b-1*, and *CiTBL1b-2*

**Figure 3:**
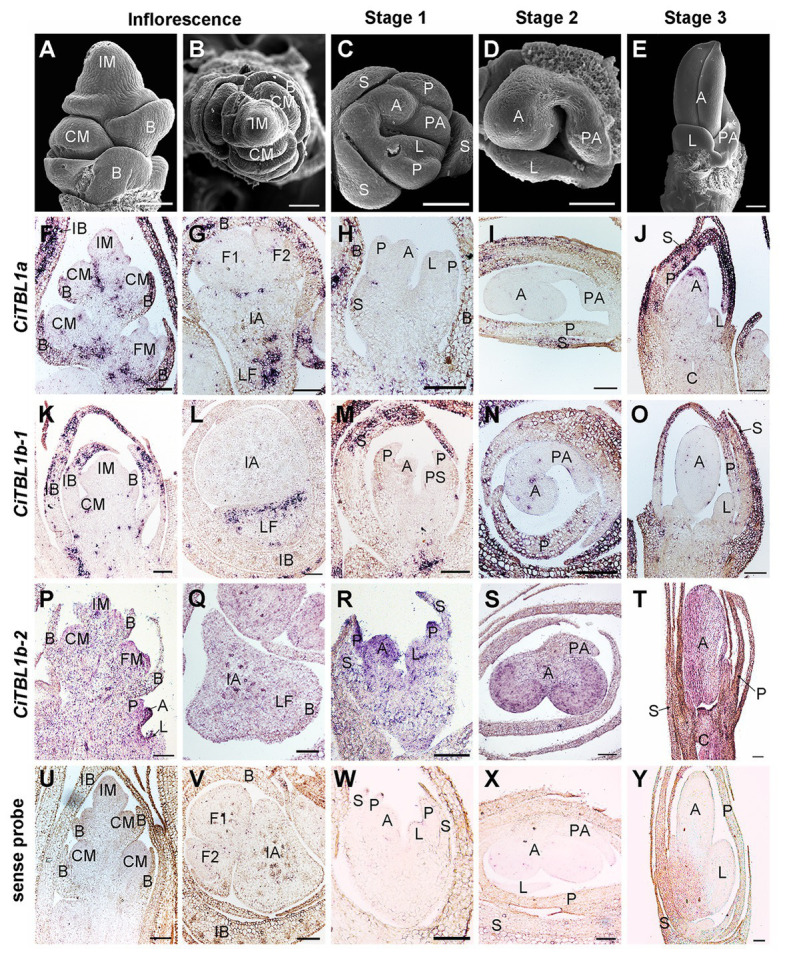
Expression patterns of *CiTBL1a*, *CiTBL1b-1*, and *CiTBL1b-2* during inflorescence and early flower developmental stages. **(A–E)** SEM microphotographs of successive developmental stages. **(F–T)** RNA *in situ* hybridizations with antisense probes of **(F–J)** *CiTBL1a*, **(K–O)** *CiTBL1b-1*, and **(P–T)** *CiTBL1b-2*. **(U–Y)** Sections hybridized with *CiTBL1b-1* sense probe as negative controls. **(G,I,L,Q,S,V,X)** were transverse sections, and all the others were longitudinal sections. IB, inflorescence bract; LF, lateral florescence. Bars = 100 μm.

**Figure 4:**
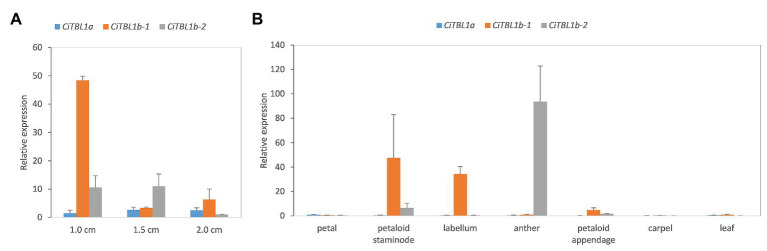
The results of qRT-PCR showing the expression patterns of *CiTBL* genes in the late floral developmental stages. Relative expression of *CiTBL1a*, *CiTBL1b-1*, and *CiTBL1b-2* in **(A)** flower bud of three different lengths and **(B)** seven different organs. Data were normalized by the relative expression of *CiTBL1a* in 1.0-cm-long flower bud in **(A)** and petal in **(B)**. Error bars of gene expression are ±1 SD from three biological replicates.

Based on previous RNA-Seq results (Tian et al., 2016), *CiTBL2* is minimally expressed in both floral primordia (FP) and differentiated flowers (DF) when compared with the other three *CiTBL* genes (Supplementary Figure S2). Thus, we focus our study on *CiTBL1a*, *CiTBL1b-1*, and *CiTBL1b-2*. Expression of *CiTBL1a*, *CiTBL1b-1*, and *CiTBL1b-2* were detected by RNA *in situ* hybridization (Figure 3) and by qRT-PCR (Figure 4) to observe their overall expression pattern during development and investigate their potential role in establishing asymmetric development of the *Canna* flower.

**Figure 5:**
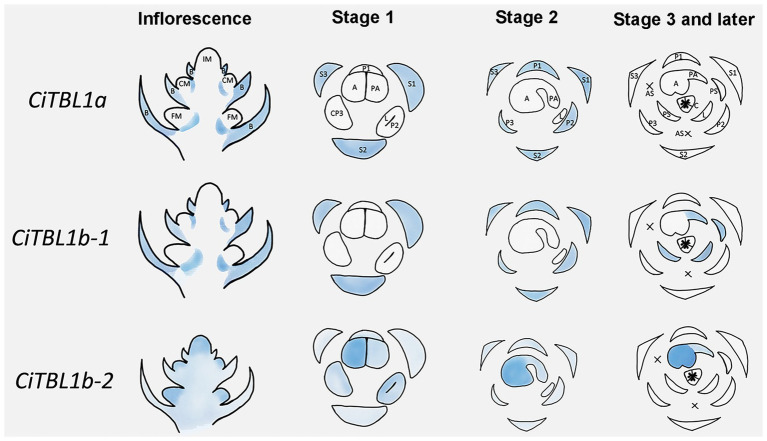
Summary for the expression patterns of *CiTBL1a*, *CiTBL1b-1*, and *CiTBL1b-2* in the young inflorescence and flowers of different developmental stages. The positions with expression are shaded in blue. The darkness of the blue shades indicates the expression level.

The results of RNA *in situ* hybridization show that *CiTBL1a* and *CiTBL1b-1* have similar expression patterns in the young inflorescence (Figures 3A,B,F,G,K,L, 5), with very strong expression in the inflorescence bracts (IB) and the primary bracts (B). The cross-sections of the inflorescence show that *CiTBL1a* and *CiTBL1b-1* are expressed on the adaxial side of the lateral florescence branches (Figures 3G,L, 5). In contrast, *CiTBL1b-2* has a broader expression pattern, with transcripts mainly accumulating in the inflorescence meristem (IM), cincinnus meristem (CM), and floral meristem (FM); no adaxial expression was detected in the lateral florescence branches (Figures 3P,Q, 5). After the common primordia differentiates, *CiTBL1b-2* is expressed in the petal whorl and inner androecium whorl. After the adaxial stamen primordium differentiates into the anther primordium and the petaloid appendage (Stage 1; Figure 3C), *CiTBL1b-2* is expressed in both anther primordium and petaloid appendage, with signal in anther stronger than in petaloid organs (Figures 3R, 5). Expression of *CiTBL1a* and *CiTBL1b-1* are not found in sepal, petal, or stamen primordia during early developmental stages (Figures 3H,M, 5). Later, however, when the anther primordium is growing rapidly (stages 2 and 3), *CiTBL1b-2* transcripts are highly enriched in the fertile anther (Figures 3S,T, 5), whereas the transcripts of *CiTBL1a* and *CiTBL1b-1* are mainly observed in developing sepals and petals (Figures 3I,J,N,O, 5).

We further examined the spatial-temporal expression of *CiTBL* genes during late floral developmental stages using qRT-PCR. The results show that the expression level of *CiTBL1a* is significantly lower in late developmental stages (stage 3 and later), whereas *CiTBL1b-1* has the highest expression in flower buds reaching 1.0 cm in length. *CiTBL1b-2* is expressed slightly more in 1.0- and 1.5-cm flower buds than it is in 2.0-cm flower buds (Figure 4A). For spatial expression patterns, the transcripts of *CiTBL1b-1* are specifically abundant in the petaloid staminode, labellum, and petaloid appendage, whereas *CiTBL1b-2* continues to be expressed primarily in the developing anther (Figures 4B, 5).

### Functional Analysis of Three *CiTBL* Genes in Transgenic *Arabidopsis* Plants

The ORF of *CiTBL1a*, *CiTBL1b-1*, and *CiTBL1b-2* were ectopically expressed in *A. thaliana* to elucidate their function during flower morphogenesis.

**Figure 6:**
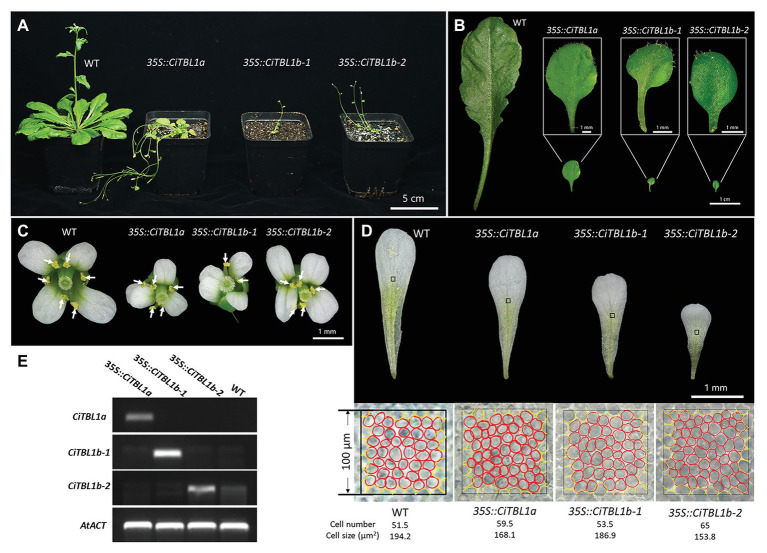
Effects of ectopic expressions of the *Canna CiTBL* genes in *Arabidopsis*. Effect of overexpressing *CiTBL1a*, *CiTBL1b-1*, and *CiTBL1b-2* on *Arabidopsis* **(A)** vegetative growth, **(B)** leaf size and shape, **(C)** flower symmetry, and **(D)** petal size. **(E)** Semi-quantitative RT-PCR results showing strong expression of *CiTBL1a*, *CiTBL1b-1*, and *CiTBL1b-2* in the flowers of T2 transgenic plants. All the stamens in **(C)** are indicated with arrows. A red circle in **(D)** represents one cell, whereas a yellow circle counted for ½ cell.

Constitutive expression of *CiTBL1a*, *CiTBL1b-1*, and *CiTBL1b-2* led to plants with relatively similar phenotypes. All the transgenic plants were dwarf and exhibited small oval-shaped leaves (Figures 6A,B), indicating that *CiTBL* genes likely inhibit vegetative growth in *Arabidopsis*. The flowers, including the individual petals, were slightly smaller in size than those of wild-type (Figures 6C,D). In addition, the induction of ectopic activity of three *CiTBL* genes in *Arabidopsis* altered the symmetry of the flower by reducing stamen number. Stamen loss was observed in all transgenic lines. For *35S::CiTBL1a* (92 flowers total), 38.1% have 5 stamens as shown in Figure 6C, and 18.5% have more severe phenotypes with only 3–4 developed stamens. For *35S::CiTBL1b-1* (106 flowers total), 25.3% of the flowers have 5 stamens (Figure 6C), whereas 16.9% have only 2–4 stamens. For *35S::CiTBL1b-2* (134 flowers total), 36.6% have 5 stamens (Figure 6C), and 11.1% have 4 stamens or fewer. In addition, growth inhibition of individual stamens is observed in flowers that still maintain six stamens. In WT *Arabidopsis*, four stamens are long and two are short. In the overexpression lines with six total stamens, either one is long and five are short or two are long and four are short. This phenomenon accounts for 2.8% of flowers in *35S::CiTBL1b-1* and 1.4% of flowers in *35S::CiTBL1b-2*. The loss of stamen primordia results in increased space in the floral meristem, and the two petals adjacent to the position of stamen loss appear to fill in the gap (Figure 6C). The overall floral symmetry changes from bilateral (WT) to pronounced zygomorphy with a single plant of symmetry.

To determine whether the small petal size of *35S::CiTBL* lines was achieved by a decrease in cell proliferation or cell expansion, the cell size of the middle region of adaxial epidermal layer in mature petals was analyzed (Figure 6D). The epidermal cells of wild-type plant petals were about 1.16, 1.04, and 1.26 times bigger than those in *35S::CiTBL1a*, *35S::CiTBL1b-1*, and *35S::CiTBL1b-2* plants, respectively. The results suggest that the effects of *CiTBL* genes on petal size is at least partially due to a decrease in cell expansion.

## Discussion

### *CYC*/*TB1*-Like Genes May Be Involved in the Formation of the Novel Organ Petaloid Staminodes in Zingiberales

In flowers of the Zingiberalean ginger families (Costaceae, Zingiberaceae, Cannaceae, and Marantaceae), petaloid staminodes account for the “showy” aspect of the floral display, taking on the role and function typically held by petals. The androecium-derived “petaloid” structures are, however, different from second-whorl petals in their appearance and epidermal cell morphology (Almeida et al., 2015), indicating that mechanisms making a petaloid structure may not be homologous across the whorls. In this research, the genomic presence and expression of endogenous *CYC*/*TB1*-like genes was studied to determine their potential role in generating petaloid structures and thereby effecting overall patterns of floral symmetry. Our results indicate *CYC*/*TB1*-like genes may play a role in the evolutionary transition from the radial, fertile stamens of the banana families to the laminar, petaloid staminodes that dominate the androecial whorls of the ginger families.

The first line of evidence is based on existing sequencing data. The *CYC/TB1*-like homolog *ZinTBL1b-1* was found exclusively in the four ginger families, which bear flowers with elaborate petaloid staminodes. We did not recover *ZinTBL1b-1* in any of the banana families, which lack a predominance of stamen-derived petaloid structures. This indicates that the presence of this particular *CYC*/*TB1*-like gene may be important in the formation of the petaloid staminodes that dominate the floral display within the ginger families.

In addition, developmental evidence for the role of *CYC*/*TB1*-like genes comes from observations of the ½-fertile stamen growth in *Canna* sp. In *Canna*, growth of the ½-fertile stamen is divided into two phases: arrest of anther growth (Figures 1D,E,I,J) and increase in petaloid appendage development (Figures 1F,G,K). We know from previous studies in other lineages that *CYC*-like genes function in stamen arrest and petal growth by affecting the rate of cell division (Luo et al., 1996; Cubas et al., 1999b; Hileman et al., 2003; Broholm et al., 2008). It is thus possible that *CYC*/*TB1*-like genes may suppress stamen fertility while promote the development of a laminar, petaloid appendage in *Canna*. Although we have not detected differential expression of any *CiTBL* genes within the *Canna* ½-fertile stamen, RT-PCR indicates that *CiTBL1b-1* is highly expressed late in development when the petaloid appendage is undergoing rapid growth (Figures 1F,G,K, 4A). In addition, *CiTBL1b-1* is expressed in all petaloid staminodes, including the labellum and the ½-stamen petaloid appendage (Figure 4B). This is further evidence that *CiTBL1b-1* in particular may be responsible for petaloid growth of the androecial members.

Finally, in the core eudicots, loss of function of C-class MADS-box genes is responsible for the transition from stamens to petals (Coen and Meyerowitz, 1991; Causier et al., 2010). Although there is no study that directly indicates the role of *CYC*/*TB1*-like genes in the origin of androecial petaloidy, studies have shown that *CYC* homologs interact with both B-class and C-class genes which are expressed in the androecial whorl. In the *Antirrhinum def* mutant, *cyc* expression disappears in the second whorl in the late developmental stage, demonstrating that *DEF* gene activity is required for later maintenance of *CYC* in this whorl (Clark and Coen, 2002). In Asteraceae, *CYC*-like genes also co-operate to regulate stamen and carpel differentiation likely through their interaction with genes controlling cell cycle and floral organ identity (Broholm et al., 2014; Fambrini and Pugliesi, 2016). In *Medicago* (Fabales), expression of all three *MtCYC* homologs is significantly upregulated in the *AG* double mutant which shows abnormality in the dorsal petal (Zhu et al., 2018). The expression pattern of *CiTBL1b-2* is similar to that of *CiAG-1*. Both *CiTBL1b-2* and *CiAG-1* were expressed slightly higher in the anther primordium than that of petaloid staminodes (Tian et al., 2016). These data indicate that symmetry genes and floral organ identity factors may act together to determine the position and final form of mature floral organs.

### Comparison of Expression Patterns of *CYC*/*TB1*-Like Genes in *Canna* and Other Monocot Species

In monocots, expression patterns of *CYC*/*TB1*-like genes appear to diverge from those reported for eudicots. For example, eudicot *CYC2* genes are typically expressed in the dorsal (adaxial) region of floral meristems, whereas in monocots the *CYC*/*TB1*-like genes are expressed on the ventral (abaxial) region in several clades with zygomorphic flowers (Yuan et al., 2009; Bartlett and Specht, 2011; Preston and Hileman, 2012; Lin et al., 2016). In the asymmetric flower of Cannaceae, the “ventral” region is distal to the main florescence axis and to the next order flower of the cincinnus on which it is borne. In this study, *CiTBL1b-1* was shown to be expressed ventrally during late stages of floral developmental; i.e., it was expressed in all petaloid staminodes (the 3/4 ventral region of the flower) and was absent from the fertile anther (the dorsal region). The ventral expression patterns of *CiTBL1b-1* and some of its monocot homologs suggest that *CYC*/*TB1*-like genes may have evolved novel functions within the monocots.

In non-monocot lineages studied to date, *CYC*-like genes are often expressed in staminodes (Luo et al., 1996; Cubas et al., 1999b; Hileman et al., 2003). However, expression of *CYC*/*TB1* has been documented in fertile stamens in monocots, including rice (*REP1*, Poacea; Yuan et al., 2009), maize (*TB1*, Poacea; Hubbard et al., 2002), *Tradescantia pallida* and *Commelina communis* (*TB1a*, Commelinaceae; Preston and Hileman, 2012), *Costus spicatus* (*CsTBL1a* and *CsTBL2*, Costaceae; Bartlett and Specht, 2011), and *Alstroemeria aurea* (*AaTCP1*, Alstroemeriaceae; Hoshino et al., 2014). Similar expression patterns have been found in this study; *CiTBL1b-2* showed strong expression throughout the development of the single fertile anther. These indicate a potentially distinct mechanism of the *CYC*/*TB1* genes in defining domains of symmetry across monocots in comparison with the well-studied eudicot model systems.

### *CiTBL*s Act as a General Repressor for Both Vegetative and Reproductive Developmental Pathways in *Arabidopsis*

The ectopic expression of all three *CiTBL* genes caused reduced vegetative growth in *Arabidopsis*, consistent with results from previous studies (Costa et al., 2005; Yang et al., 2012; Juntheikki-Palovaara et al., 2014; Liu et al., 2014) and indicating conserved function of *CYC*/*TB1*-like genes in vegetative development. However, heterologous overexpression of various *CYC*/*TB1*-like genes in *Arabidopsis* produces differential changes in flower development. Overexpression of *Antirrhinum CYC* in *Arabidopsis* produced larger flowers with larger petals (Costa et al., 2005), whereas overexpression of *Gerbera CYC2* genes, *Primulina CYC1C* and *CYC1D*, and *Canna CiTBL* genes (this study) all led to smaller flowers (Yang et al., 2012; Juntheikki-Palovaara et al., 2014; Liu et al., 2014), indicating that the function of *CYC*-like genes on reproductive growth varies. In this study, we also found reduced number of stamens in the transgenic plants, a phenomenon which has not been reported in previous studies. The stamen loss caused modification of floral symmetry in transgenic *Arabidopsis* plants. Interestingly, a similar phenotype was found in *Arabidopsis* plants with overexpressed *Chrysanthemum CmWUS*, indicating a potential interaction between *WUSCHEL*- and *CYC2*-like genes in regulating flower development (Yang et al., 2019). Due to the heterologous genetic background of *Arabidopsis* and *Canna*, we cannot draw a comprehensive conclusion as to the functions of *CiTBL* genes during *Canna* flower development. Genetic knock down or homologous overexpression strategies are needed to investigate the exact functions of *CYC*/*TB1*-like genes during the development of the asymmetric flower of *Canna*.

## Conclusion

Asymmetry is a very unusual type of flower form not often included in studies of floral symmetry. *Canna* bears conspicuous asymmetric flowers due to the presence and distribution of androecial petaloidy within the two androecial whorls. In this study, four *CYC*/*TB1* homologs were isolated and their expression was analyzed to reveal their potential roles in the establishment of asymmetry of the wild-type *Canna* flower. The results indicate that *CiTBL* genes are probably involved in asymmetric flower development given their asymmetric expression both early and late during floral development. Notably, *ZinTBL1b-1* was expressed in the all androecial petaloid structures, suggesting it may play a role in the morphogenesis of petaloid staminodes. Heterologous overexpression of *CiTBLs* acts as a general repressor for both vegetative and reproductive developmental pathways in *Arabidopsis*, and alters floral symmetry by arresting the growth of stamens. Overall, this study provides insights for revealing the developmental mechanisms underlying the formation of asymmetric flowers.

## Data Availability Statement

The datasets presented in this study can be found in online repositories. The names of the repository/repositories and accession number(s) can be found in the article/Supplementary Material.

## Author Contributions

XT and JL conceived and designed the experiments. QY performed the experiments and wrote the manuscript. CL and CS edited the manuscript. JL coordinated all tasks. All authors contributed to the article and approved the submitted version.

### Conflict of Interest

The authors declare that the research was conducted in the absence of any commercial or financial relationships that could be construed as a potential conflict of interest.

## Footnotes

^1^https://banana-genome-hub.southgreen.fr/
