# Supplementary material for: Expression and Function Studies of CYC/TB1-Like Genes in the Asymmetric Flower Canna (Cannaceae, Zingiberales)
Source: Front Plant Sci. 2020 Dec 4;11:580576. doi: 10.3389/fpls.2020.580576 (PMC7746682; doi:10.3389/fpls.2020.580576)
Supplement: Supplementary file 1 [file Image_1.pdf]

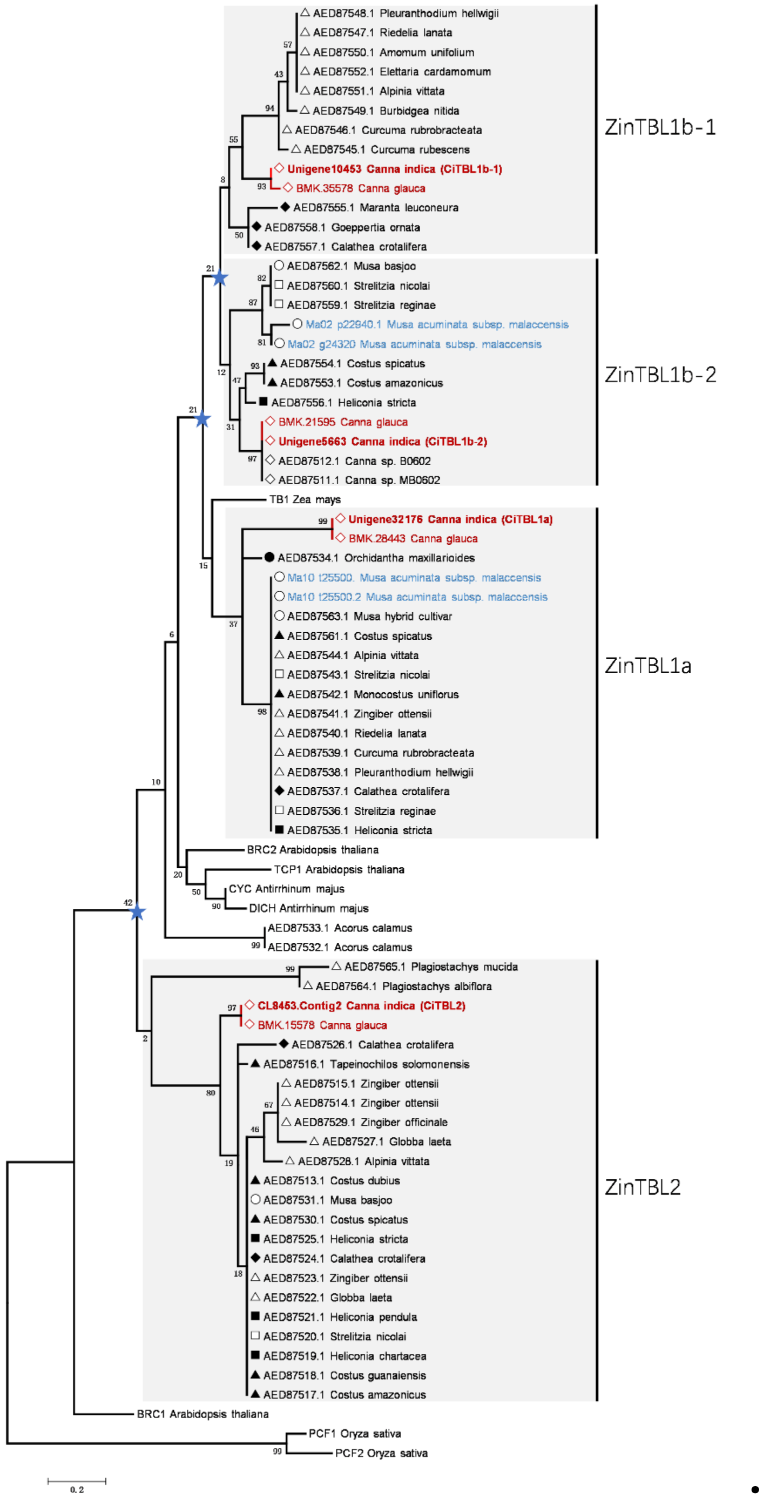

Supplementary Figure 1. Maximum likelihood tree of *CYC/TB1*-like genes in Zingiberales. Red color indicates *CYC/TB1* genes from *C. indica* and *C. glauca*. Blue color indicates *CYC/TB1* genes from the banana genome. Stars indicate gene duplication events.
