# Supplementary material for: Expression and Function Studies of CYC/TB1-Like Genes in the Asymmetric Flower Canna (Cannaceae, Zingiberales)
Source: Front Plant Sci. 2020 Dec 4;11:580576. doi: 10.3389/fpls.2020.580576 (PMC7746682; doi:10.3389/fpls.2020.580576)
Supplement: Supplementary file 2 [file Image_2.pdf]

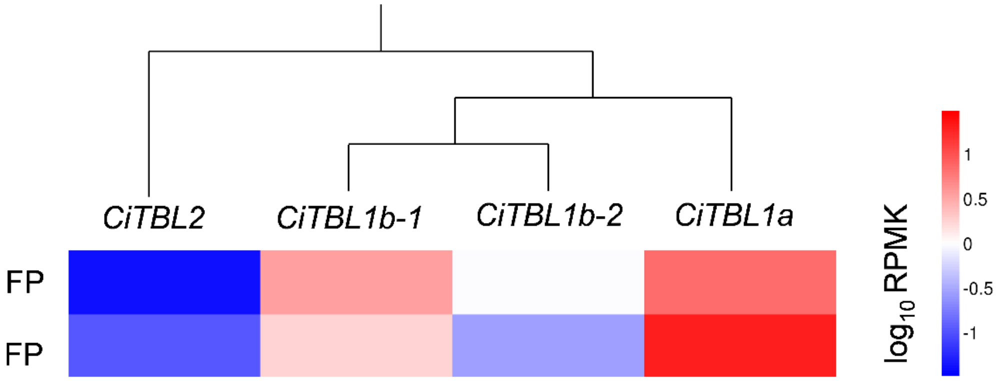

Supplementary Figure 2. Expression heatmap of *CiTBL* genes. FP, floral primordia; DF, differentiated flower.
