# Supplementary material for: Expression and Function Studies of CYC/TB1-Like Genes in the Asymmetric Flower Canna (Cannaceae, Zingiberales)
Source: Front Plant Sci. 2020 Dec 4;11:580576. doi: 10.3389/fpls.2020.580576 (PMC7746682; doi:10.3389/fpls.2020.580576)
Supplement: Supplementary file 3 [file Table_1.docx]

**Supplementary Table 1.** Primers used in this study.

| **Primer ID** | **Sequence (5’ 🡪 3’)** | **Usage** |
| --- | --- | --- |
| Unigene32176_AllF | TCCCTAACTCCTCAACCCTC | *CiTBL1a* full-length cDNA cloning |
| Unigene32176_AllR | TGCAATAGCAACACAGTTTCAT |  |
| Unigene10453_AllF | CTCAACCCACCACCTCACAT | *CiTBL1b* full-length cDNA cloning |
| Unigene10453_AllR | GAACAGCTCCTTTGGTGTAGC |  |
| Unigene5663_AllF | AGGCGCAAATATTCCACATGC | *CiTBL2* full-length cDNA cloning |
| Unigene5663_AllR | ACGTTCTCCCCCTTTAACATTAT |  |
| Unigene32176F(A1F) | TCTAGACTCCCTTTCGGTTCTGGCTC | *CiTBL1a* probe cloning |
| Unigene32176R(A1R) | AAGCTTGCGATCTCTAGGCCCAGTTG |  |
| Unigene10453F(C1F) | GGATCCTAGCCAAGGCTGCTTACGAG | *CiTBL1b-1* probe cloning |
| Unigene10453R(C1R) | TCTAGAGTGTTGCCCATGTGGTTGTG |  |
| Unigene5663F(B2F) | CTCGAGCGGAATCAAAACCTGTCCGC | *CiTBL1b-2* probe cloning |
| Unigene5663R(B2R) | GGATCCAAGTCATTGCTCGACCTCGG |  |
| 3rd-TBL1a-qPCR-F | GACCGTAAGGGCAAATCCGA | *CiTBL1a* qRT-PCR |
| 3rd-TBL1a-qPCR-R | CATCACACCGGAGGCATTCT |  |
| TBL1b-1-qPCR-F | CTTGTACGGCAAACCAGTGC | *CiTBL1b-1* qRT-PCR |
| TBL1b-1-qPCR-R | TCAGTGATTGGTGTGGCTCC |  |
| TBL1b-2-qPCR-F | TTGGACATCGTGAGCAACCA | *CiTBL1b-2* qRT-PCR |
| TBL1b-2-qPCR-R | CACAGCAAGATATGCACGGC |  |
| PP2A-F | TCACTGGTTAGTGCTTGGCA | *PP2A* qRT-PCR |
| PP2A-R | AGTTGCAGGTAAACGCCTCA |  |
| F-Actin-c74912.graph_c0 | AACAGGAGTTGGAGACTGCC | *Actin* qRT-PCR |
| R-Actin-c74912.graph_c0 | AGGACCTCTGGGCACCTAAA |  |
| TBL1a-ZB-F | CTCTTGACCATGGTAATGTTACCATTCCCTAACTCCT | Construction of *CiTBL1a* transgenic plants |
| TBL1a-ZB-R | CTCCTTTACTAGTCATCATTCCATGTCAGCGTGGT |  |
| TBL1b-1-ZB-F | CTCTTGACCATGGTAATGCTGTCATTTCCTGATCC | Construction of *CiTBL1b-1* transgenic plants |
| TBL1b-1-ZB-R | CTCCTTTACTAGTCATTACCTGGAGTAGAATGAATCC |  |
| TBL1b-2-ZB-F | CTCTTGACCATGGTAATGCATCTCTTTCATGAACAAG | Construction of *CiTBL1b-2* transgenic plants |
| TBL1b-2-ZB-R | CTCCTTTACTAGTCATTAATTTGGGTAGTCGTTGAAG |  |
| F206 | GGATGACGCACAATCCCACTA | Positive cloning of pCAMBIA1302 identity |
| R206 | TTCACCCTCTCCACTGACAGA |  |
| AtActin-F | TGGCATCAYACTTTCTACAA | Semi-quantitative RT-PCR analysis in transgenic *Arabidopsis* plants |
| AtActin-R | CCACCACTDAGCACAATGTT |  |
